# Non-traditional risk factors of progression of chronic kidney disease in adult population: a scoping review

**DOI:** 10.3389/fmed.2023.1193984

**Published:** 2023-06-02

**Authors:** Diana Lorena Cisneros-García, Elena Sandoval-Pinto, Rosa Cremades, Adrián Ramírez-de-Arellano, Mariana García-Gutiérrez, Roberto Martínez-de-Pinillos-Valverde, Erick Sierra-Díaz

**Affiliations:** ^1^Departamento de Salud Pública, Centro Universitario en Ciencias de la Salud, Universidad de Guadalajara, Guadalajara, Jalisco, Mexico; ^2^Departamento de Biología Celular y Molecular, Centro Universitario de Ciencias Biológico Agropecuarias, Universidad de Guadalajara, Guadalajara, Jalisco, Mexico; ^3^Departamento de Microbiología y Parasitología, Centro Universitario en Ciencias de la Salud, Universidad de Guadalajara, Guadalajara, Jalisco, Mexico; ^4^Instituto de Investigación en Ciencias Biomédicas, Centro Universitario de Ciencias de la Salud, Universidad de Guadalajara, Guadalajara, Mexico; ^5^Centro Metropolitano de Atención de la Diabetes Tipo 1, Secretaría de Salud Jalisco, Guadalajara, Jalisco, Mexico; ^6^División de Trasplantes, UMAE Hospital de Especialidades Centro Médico Nacional de Occidente del IMSS, Guadalajara, Mexico; ^7^Departamentos de Clínicas Quirúrgicas y Salud Pública, Centro Universitario de Ciencias de la Salud, Universidad de Guadalajara, Guadalajara, Mexico; ^8^División de Epidemiología, UMAE Hospital de Especialidades Centro Médico Nacional de Occidente del IMSS, Guadalajara, Mexico

**Keywords:** end-stage kidney disease, end-stage renal failure, chronic kidney failure, chronic renal failure, non-traditional, unknown causes

## Abstract

Chronic kidney disease (CKD) has become a public health concern over the last several years. Nowadays developed countries spend around 3% of their annual health-care budget on patients with CKD. According to the scientific community the most remarkable risk factors for CKD are diabetes and hypertension. Unknown CKD etiology has been reported as a global phenomenon including uncommon risk factors such as: dehydration, leptospirosis, heat stress, water quality, and others. This study aims to report non-traditional risk factors for ESRD based on a scoping review methodology. The scoping review methodology described by Arksey and O’Malley was used by performing an extensive review of the information. A total of 46 manuscripts were reviewed. The non-traditional ESRD risk factors are depicted based on six categories. Gender and ethnicity have been considered as risk factors for ESRD. Erythematous systemic lupus (ESL) is reported as an important risk factor for ESRD. Pesticide use has been an significant risk factor due to its effects on human and environmental health. Some compounds commonly used in homes against insects and plants are related to ESRD. Congenital and hereditary diseases in the urinary tract have been studied as a cause of ESRD in children and young adults. End-stage renal disease is a major concern for public health on a global level. As it can be seen, non-traditional risk factors are several and have different etiologies. It is necessary to put the issue on the table and add it to the public agenda in order to find multidisciplinary solutions.

## 1. Introduction

Chronic kidney disease (CKD) has become a public health concern over the last several years. It is a major problem, with some developed countries spending around 3% of their annual health-care budget on management every year (1). Before the SARS-CoV-2 pandemics, the reported prevalence at the international level was 13.4%, and around 4 to 7 million people at end-stage renal disease (ESRD) who needed replacement therapy (2).

According to the scientific community the most remarkable risk factors for CKD are diabetes and hypertension. However, some other risk factors should be taken into account such as: HIV-associated nephropathy, viral infections (dengue) or break-bone fever, malaria, leishmaniasis, leptospirosis and others (1). Research from 2015 reported that more than a million deaths, and around 19 million disabilities were related to reduced glomerular filtration rates secondary to cardiovascular diseases (1, 3, 4). Inflammation is another well-studied condition related to CKD. Some authors have described that an inflammatory stage could work as a maladaptive and uncontrolled disorder in CKD patients and is associated with cardiovascular disease (5–7).

The unknown etiology CKD has been reported as a global phenomenon including uncommon risk factors such as: dehydration, leptospirosis, heat stress, water quality and others (8). However, the number of uncommon causes of CKD has not been limited, especially due to changes in global health after the SARS-CoV-2 pandemics. Their long term effects on kidney health are not known at the moment, and several years shall pass after gaining enough knowledge about it.

In Mexico, the problem is not minor. The prevalence of albuminuria in children in some rural areas is reported to be over 40% and the frequency of deaths secondary to ESRD is near 15 per 1,000 among adult inhabitants (9). Other studies in Mexican rural areas have reported similar results (10, 11). As previously mentioned, the number of unknown ESRD causes are several but not reported at all.

This study aims to report on the non-traditional risk factors for CKD progression to ESRD using a scoping review methodology.

## 2. Materials and methods

The methodology described by Arksey and O’Malley (12) was used performing an extensive review of the information. The literature review question was defined using key words included in MESH terms from PubMed, which was the only source for information. The key words that were included were: end-stage renal disease, end-stage kidney disease, end-stage renal failure, chronic kidney failure, chronic renal failure, non-traditional, unknown, causes, diabetes and hypertension; applying Boolean operators. The information was not limited with regard to time. The number of articles resulting from the previously mentioned key word combination was 707. Key words and number of hints are described in Supplementary Table S1.

A total of 46 manuscripts were reviewed. Selection criteria included: The English language, the full text, and quantitative observational studies (cross-sectional, case–control and cohort studies) in the adult population. Reviews, clinical trials, case reports, qualitative and studies including children were not included for review.

The review and selection of manuscripts were performed during a 4-month period by pairs. The selected manuscript was systematized in an excerpt matrix (worksheet) that included: the lead author, year and place, objective, study design, sample, variables, statistical analysis, and results. Supplementary Table S2 summarizes the characteristics of the studies included.

As diabetes and hypertension are mainly the most common ESRD risk factors, and all manuscripts which included such topics were excluded. Studies performed on children were also excluded.

## 3. Results

As previously mentioned, diabetes mellitus and hypertension are the most common risk factors for ESRD. However, in several cases, the etiology is unknown (13–17). Globally, ESRD is a public health concern affecting people’s quality of life. In order to carry out some strategies to prevent its harmful effects on the health of young adults, all possible risk factors must be well documented. The non-traditional ESRD risk factors are depicted based on six categories.

### 3.1. Sociodemographic characteristics (gender, race, rurality and area deprivation)

Gender and ethnicity have been considered as risk factors for ESRD. Some researchers concluded that the risk is higher among males (18–21). Regarding ethnicity, African-American (AA) is defined as a risk factor (HR 3.0 CI95: 2.58–3.54) among United States residents (21). The relative risk for ESRD for AA compared to Caucasians was 2.86 (CI95: 2.35–3.47) (20). The same researchers concluded that the risk of ESRD is twice as great in rural areas residents compared to people who are living in urban areas (19). Crews et al., reported that low income is related to ESRD (HR 3.75 95CI 8.62–8.64). Middle income level subjects showed lower risk (1.0 95CI 1.01–1.06) and those individuals with high incomes reported HR values <1 (22). Income and ESRD is not a novel study topic since their relationship was reported in 2008 by Bello et al., who reported an odds ratio value of 11.90 (95IC 8.5–16.7), whereas the OR for high income was 1.43 (95IC 1.01–2) (23).

### 3.2. Non-transmissible diseases

ESRD is related to chronic diseases. Among non-transmissible diseases there are several related causes which are not always reported as an important risk factor for this global health issue. Systemic Lupus Erythematosus (SLE) is reported as an important direct cause of ESRD in South Korea (HR 9.84 CI95 8.10–11.8) and Eastern United States (Atlanta, Georgia) (HR 6.7 95CI 2.6–16) (24, 25). Complement factors such as high levels of C3 were reported as a risk factor for ESRD in a retrospective cohort of 338 SLE patients in Germany (RR 3.0 95CI 1.5–6) (26). Wunnenburger et al. reported that SLE risk variant (*TNXB*) is associated with CKD attributed to type 1 diabetes mellitus (*p* < 0.01), and IgA nephropathy (*HLA-DRB1*) was related to granulomatosis with polyangiitis (*p* < 0.01) (27).

Obesity has been related to kidney diseases without differences regarding gender (28, 29). The risk for ESRD is gradually increasing in individuals who are overweight to grade 3 obesity from 2.2 (CI95 1.7–2.7) to 6.5 (CI95 4.6–9.3). Indeed, differences between people who are overweight and grade 1 obesity are remarkable (HR 3.6 CI95 2.8–4.6). Living kidney donors have an overall HR of 1.86 of developing ESRD and, the HR value increased twice when donors are obese (17). It is important to mention that those obese individuals who were included in the previous studies had neither diabetes nor hypertension. Other authors from Austria and Finland that included around 29,000 individuals reported that a body mass index (BMI) between 25 to 30 confers a RR of 1.58 for ESRD. Those with BMI over 31 showed a higher risk (RR 2.5 CI95 1.6–4.4) (30, 31).

Chronic disease associated anemia that is non-malignant related has been reported as a risk factor for ESRD (RR 2.81 and HR 1.55) (31), whereas other authors from Pakistan reported that there is no causal relationship between both diseases (20).

### 3.3. Environmental factors

Pesticides have been widely used and have affected the health of both humans and the environment. Some compounds commonly used in homes against insects and plants are related to ESRD. Hsu *et al*, reported that exposure to some toxic household substances in California, United States is a risk factor for ESRD (HR 1.78 CI95 1.3–2.3) (21). Other compounds used in agricultural activities have been associated with ESRD. The higher HR reported values were for metalaxyl (HR 1.92 CI95 1.01–3.66), atrazine (HR 1.51 CI95 1.1–2.06), pendimethalin (HR 2.15 CI95 1.23–3.77), metolachlor (HR 1.53 CI95 1.08–2.18), alachlor (HR 1.56 CI95 1.12–2.18) and paraquat (HR 2.23 CI95 1.18–4.21) (32). Chemical compounds such as asbestos, cement, or grain dust were reported in 2009 as risk factors for ESRD, with HR values of 1.77 (CI95 1.34–2.33) and chemical solvents 1.61 (1.37–1.90). The same authors (21) reported extremely high temperature exposure as a risk factor (HR 1.73 CI95 1.34–2.24). Lead compounds are historically related to several diseases and ESRD is not the exception. Serum levels around 3.5 and 3.9 mmol/L increased the risk for disease (HR 1.39 CI95 1.13–1.70) (33). Other authors reported an odds ratio of 1.01 (CI95 1.0–1.02) after exposure (34). Stressful working conditions were reported as a risk factor for ESRD by Aksoy et al. in 2020 in Turkey. Researchers performed a cross-sectional study that included 258 individuals. The conclusion was that work stress represents an important issue in patients before kidney transplantation (OR 5.86 CI95 2.21–15.52) (35). Tobacco smoking is one of the most remarkable public health issues globally. Its relationship with ESRD has been documented. The HR values reported in 2015 and 2021 were 1.30 (CI95 1.04–1.63) and 1.87 (CI95 1.1–3.19) respectively (17, 36).

### 3.4. Congenital and mineral metabolism disorders

Congenital and hereditary diseases of the urinary tract have been studied as a cause of ESRD in children and young adults. Ureteropelvic junction obstruction is the most common congenital abnormality diagnosed in children. However, in many cases, this congenital abnormality is diagnosed in adults being unilateral combined with other chronic diseases or bilateral in some cases causing ESRD (37). Other diseases such as Alport syndrome and MYH9 mutations were reported as the cause of CKD (38).

Kidney stone disease is not a common cause of ESRD since new treatments allow for the management of the problem and yields excellent results (endourology procedures) (39–41). However, the risk of ESRD with the presence of kidney stone disease increases in a remarkable way (OR 16.1 CI95 5.7–45.4) (42).

### 3.5. Drug use

The use of non-steroidal anti-inflammatory drugs (NSAIDs) has been related to the risk of inducing kidney failure. The use of this type of medication should be controlled in patients who have some type of kidney failure in order to avoid more damage. However, the risk for CKD in healthy patients is not well known. Some observational studies have reported data related to the risk of CKD in long term users of oxicams (OR 1.74 CI95 1.20–2.54), meloxicam (OR 1.98 CI95 1.01–3.87), piroxicam (OR 1.95 CI95 1.19–3.21) and ketorolac (OR 2.54 CI95 1.45–4.44) (43). As it can be seen, the four compounds showed a significant relationship between study variables.

Another study reported that CKD was associated with regular NSAIDs users. The research adjusted the OR values for sex and age, resulting in eGFR <60 mL/min per 1.73 m2 (OR 1.51 CI95 1.13–2.02), and albuminuria (OR 1.31 CI95 1.07–1.59). Same authors identified that the cumulative time of NSAID intake and kidney damage for those who took them for more than 48 months had an eGFR <60 mL/min per 1.73 m2 (OR 2.36 CI95 1.28–4.37) (44). A previous study performed in 2005 showed a non-significant relationship among the chronic use of NSAIDs (OR 1.22, CI95 0.89–1.66). The research investigated the uses of analgesics for cardiovascular conditions, headaches, joint pain, and the musculoskeletal system (45). CKD patients using acetaminophen, aspirin, COX-2 inhibitors, and other NSAIDs had an increased risk of progression to ESRD with multivariable adjusted HR of 2.92 (CI95 2.47–3.45), 1.96 (CI95 1.62–2.36), 1.54 (CI95 1.08–2.20) and 1.56 (CI95 1.32–1.85), respectively (46).

In subjects with a mean glomerular filtration rate of 60–89 mL/min/1.73 m2, COX-2 inhibitor users had a 25% increased risk of rapidly progressing kidney disease (OR 1.25 CI95 1.05–1.47) and traditional users of NSAIDs a 29% higher risk (OR 1.29, CI95 1.02–1.63) compared to non-users of NSAIDs (47).

The use of second-generation antipsychotics (SGA) has also been a debatable issue regarding the development of ESRD. The literature has shown that the general risk for having used an SGA at some moment in their life yielded an OR of 1.24 (CI95 1.12–1.37). The corresponding OR for current use was 1.26 (CI95 1.12–1.42). The risk was more pronounced for clozapine (OR 1.81 CI95 1.22 to 2.69) followed by olanzapine (OR 1.41 CI95 1.19–1.65) and quetiapine (OR 1.28 CI95 1.17–1.42) (48).

Moreover, the use of illegal drugs such as heroin or other opiates, cocaine, amphetamines, tranquilizers, psychedelics and cannabis products (marijuana or hashish), has also been reported as an important risk factor for the development of CKD. In the United States a case–control study was carried out in 716 patients aged 20–64 years that identified the use of cannabis-derived products more than 100 times in their entire lives, which was associated with ESRD (OR 2.5 CI95 1.4–5.0), and in terms of lifetime use of amphetamines greater than 100 times was also associated with an increased risk of ESRD (OR 4.9 CI95 1.2–43.5). Regarding the use of cocaine, it was determined that the dose–response relationship of this increases the risk of End-Stage Renal Disease (ESRD) in people who used it less than 100 times in their lives (OR 2.6 IC951.2–6.5), compared to those people who used it more than 100 times in their lives (OR 8.7 CI95 2.2–75.3). Heroin use showed a strong and statistically significant increased risk for ESRD (OR 20.3 CI95 3.6-infinite) (49).

### 3.6. Others

The novel culture of organ and tissue transplantation has played an important role in public health. The facts concerning sharing an organ is different regarding time and place. Third world countries such as Mexico and Brazil have a remarkable preference about living kidney donors. Conversely, countries like the United States and Spain have a greater tendency for cadaveric donors. An important issue regarding living donors (kidney) is the risk of developing ESRD, a fact that has been documented. Massie et al., performed a retrospective cohort that included 71,468 kidney donors. The cumulative incidence of ESRD 15 years after nephrectomy was 11.7 per 10,000 (average GFR after 6 months 70 mL/min/m2). It concluded that GFR 6 months after nephrectomy is an independent variable and potential predictor for ESRD in living kidney donors (50). Radioactive emanations have been investigated and international literature has shown some level of association with ESRD, specifically after cancer management radiotherapy (39). Lange et al., reported a retrospective cohort that included 9,237 with a history of radiotherapy for Wilms tumor management. The reported risk for ESRD in the study group was 4.2 (HR, CI95 1.6–10.7) (51).

## 4. Discussion

End Stage Renal Disease is a major concern for public health at a global level. As it can be seen, non-traditional risk factors are numerous and have different etiologies. The main risk factors reported globally have already been described (diabetes and hypertension). However, risk factors should be classified according to geographical and economic conditions. CKD screening has been recommended as a cost-effective strategy in high-risk patients allowing starting early management of the prevention of progression and identification of specific conditions. These types of early actions that work as preventive medicine are considered as the key for better public health practices that provide some opportunities for improved patient access to better attention and improved outcomes (52). Screening costs may vary depending on the country. In developed countries (high income) the cost per year is estimated between 50,000 to 150,000 USD. In Mexico the cost for a basic blood and urine tests is around 25 to 250 USD (53). This type of screening is good enough to detect early kidney damage in every patient including groups of people that are looking for an annual medical checkup. In 2014, Komenda et al. reported a worldwide review regarding costs of proteinuria and glomerular filtration rate in different groups of patients including the general population, diabetics, and hypertensive patients. The reported cost was between $100,253 to $109,912 USD. The authors concluded that CKD screening is cost-effective in patients with identifiable risk factors (54).

Although there is an idea of how to counteract ESRD for these two most common causes, it is a matter of concern that in many of the studies the etiology of the disease is still not clear. Consequently, there is insufficient knowledge regarding how to contribute with a solution.

This health problem, as previously mentioned, has an impact on everyone, but specifically in Mexico there are endemic areas of the disease, such as Jalisco and Aguascalientes, which have high incidences in children, adolescents, and young adults (55, 56).

It is important for health professionals to attend to these patients from early detection since the costs in the different levels of care for kidney patients are high, aside from how significantly their quality-of-life decreases, both for patients and their family members involved in the process (57–59).

For those individuals that have been diagnosed with ESRD, it must be considered that their disease will last a lifetime, so they will have to search for some other replacement medical treatments that are currently available, such as: peritoneal dialysis, hemodialysis and transplantation (60, 61). In Mexico, those who have social security through insurance policies associated with their school or work will have access to one of the first two treatments (61). However, access to a kidney transplant will be influenced by the search for a donor by the kidney patient himself/herself, which often complicates this process, since a living donor must be found among his/her own relatives or acquaintances, instead of a search for a cadaveric donor, as is commonly done in other countries (61, 62).

In Mexico, receptor candidates for kidney transplant, need to perform a series of studies and a biopsy to determine the cause of kidney failure and the current damage to the kidney (56, 61). This practice is expensive, but it could contribute to the problem that has developed when this paper was written, in order to identify some causes that have been neglected, which are important in the development of ESRD. The public health system should propose alternatives that help to identify these factors that would undoubtedly assist in explaining a portion of this great problem (58).

Lastly, it’s important to mention the role of diagnosis before kidney transplantation. As previously mentioned, living kidney donors are at risk of ESRD (incidence 11.7 per 10,000) (50), and several times the cause can be prevented based on medical history data such as family members with diabetes, hypertension, and other CKD risk factors. Candidates to become living kidney donors must be studied in depth to avoid diabetes and other chronic causes related to kidney disease. Several reports have shown the importance of graft biopsies for prognosis in children and adults (60, 63–65). However, information about kidney biopsy as a diagnostic tool before transplantation is scarce. The same applies to medical programs that propose the probability of graft loss secondary to that of primary diseases, or other factors such as follow-ups and attachment therapy issues. In 2012, Sellarés et al. reported that non-attachment therapy was the cause of renal transplant failure in 26 of 315 patients. Antibody-mediated rejection and T cell-mediated rejection represented 18 and 9% of kidney graft failure, respectively (66). Recurrent glomerulonephritis is reported in 10 to 20% after kidney transplantation and around 50% have a graft lost. Even follow up is in long term, the cause is generally unknown and represents the third most common cause of graft failure (67). It is clear that kidney transplant programs worldwide are ambitious, and their main intention is to improve patients’ lives. However, some programs are focused on numbers only. Several kidney transplant programs must restate the way in which they will act. Public policy transfer cannot be applied in the same way in different countries. Although there are international guidelines for kidney transplant protocols (68), local guidelines must be adapted in order to improve selection criteria and to avoid short term complications and a second transplant.

The aim of this review was to find as much information as possible in order to show a general panorama of non-traditional causes for CKD progression. However, the information about the topic is extensive and dynamic. Although we have tried to be as exhaustive as possible, there are several non-traditional causes for CKD that are not included in this paper. Perhaps, the number of unknown causes of CKD is beyond the modern knowledge and ready to be studied in future reviews.

## 5. Conclusion

End Stage Renal Disease is an international major concern for public health. The number of deaths per year is alarming and is increasing gradually, owing to an increasing prevalence of diabetes mellitus and other cardiovascular diseases. Epidemiological transition in developing countries should be taken into account since infectious diseases, environmental exposures, and other risk factors for ERSD increase the burden of disease. This fact affects not only public health policy. In general terms, the problem has the capability to seriously affect public policy at several levels. It is necessary to put the issue on the table and add it to the public agenda in order to find multidisciplinary solutions.

## Author contributions

DC-G and ES-D: conceptualization, project administration, and writing-original draft preparation. ES-P, RC, AR-d-A, MG-G, and RM-d-P: investigation and writing-review. ES-D: editing. All authors participated directly in the manuscript, the investigation, writing the original draft preparation, the writing of the review, and read and agreed to the published version of the manuscript.

## Conflict of interest

The authors declare that the research was conducted in the absence of any commercial or financial relationships that could be construed as a potential conflict of interest.

## Publisher’s note

All claims expressed in this article are solely those of the authors and do not necessarily represent those of their affiliated organizations, or those of the publisher, the editors and the reviewers. Any product that may be evaluated in this article, or claim that may be made by its manufacturer, is not guaranteed or endorsed by the publisher.

## References

[1] Luyckx VA Tonelli M Stanifer JW. The global burden of kidney disease and the sustainable development goals. Bull World Health Organ. (2018) 96:414–422D. doi: 10.2471/BLT.17.206441, PMID: 29904224 PMC5996218

[2] Lv JC Zhang LX. Prevalence and disease burden of chronic kidney disease. Adv Exp Med Biol. (2019) 1165:3–15. doi: 10.1007/978-981-13-8871-2_1 31399958

[3] GBD 2015 DALYs and HALE Collaborators. Global, regional, and national disability-adjusted life-years (DALYs) for 315 diseases and injuries and healthy life expectancy (HALE), 1990-2015: a systematic analysis for the global burden of disease study 2015. Lancet. (2016) 388:1603–58. doi: 10.1016/S0140-6736(16)31460-X 27733283 PMC5388857

[4] GBD 2015 Mortality and Causes of Death Collaborators. Global, regional, and national life expectancy, all-cause mortality, and cause-specific mortality for 249 causes of death, 1980-2015: a systematic analysis for the global burden of disease study 2015. Lancet. (2016) 388:1459–544. doi: 10.1016/S0140-6736(16)31012-1 27733281 PMC5388903

[5] Cobo G Lindholm B Stenvinkel P. Chronic inflammation in end-stage renal disease and dialysis. Nephrol Dial Transplant. (2018) 33:iii35–40. doi: 10.1093/ndt/gfy175, PMID: 30281126 PMC6168801

[6] Jankowska M Cobo G Lindholm B Stenvinkel P. Inflammation and protein-energy wasting in the uremic milieu. Contrib Nephrol. (2017) 191:58–71. doi: 10.1159/000479256, PMID: 28910791

[7] Galli F. Protein damage and inflammation in uraemia and dialysis patients. Nephrol Dial Transplant. (2007) 22:v20–36. doi: 10.1093/ndt/gfm294, PMID: 17586842

[8] Lozano-Kasten F Sierra-Diaz E De Jesus Celis-de la Rosa A Margarita Soto Gutiérrez M Aarón Peregrina Lucano A. Research group on social and environmental determinants in childhood. Prevalence of albuminuria in children living in a rural agricultural and fishing subsistence Community in Lake Chapala, Mexico. Int J Environ Res Public Health. (2017) 14:1577. doi: 10.3390/ijerph14121577 29240709 PMC5750995

[9] Gongora J Serna F Gutierrez M Perez C Hernández E Ron O. Prevalencia de enfermedad renal crónica en niños de Aguascalientes, México. Salud Pública Mex. (2008) 50:436–7. doi: 10.1590/S0036-36342008000600002, PMID: 19058410

[10] Cárdenas-González M Osorio-Yáñez C Gaspar-Ramírez O Pavković M Ochoa-Martínez A López-Ventura D. Environmental exposure to arsenic and chromium in children is associated with kidney injury molecule-1. Environ Res. (2016) 150:653–62. doi: 10.1016/j.envres.2016.06.032, PMID: 27431456 PMC5003729

[11] Arksey H O’Malley L. Scoping studies: towards a methodological framework. Int J Soc Psychiatr Res Methodol. (2005) 8:19–32. doi: 10.1080/1364557032000119616

[12] Malekmakan L Haghpanah S Pakfetrat M Malekmakan A Khajehdehi P. Causes of chronic renal failure among Iranian hemodialysis patients. Saudi J Kidney Dis Transpl. (2009) 20:501–4. PMID: 19414964

[13] Safarikova M Stekrova J Honsova E Horinova V Tesar V Reiterova J. Mutational screening of inverted formin 2 in adult-onset focal segmental glomerulosclerosis or minimal change patients from the Czech Republic. BMC Med Genet. (2018) 19:147. doi: 10.1186/s12881-018-0667-9 30126379 PMC6102913

[14] Elsharif ME Elsharif EG. Causes of end-stage renal disease in Sudan: a single-center experience. Saudi J Kidney Dis Transpl. (2011) 22:373–6. PMID: 21422650

[15] Banaga AS Mohammed EB Siddig RM Salama DE Elbashir SB Khojali MO. Causes of end stage renal failure among haemodialysis patients in Khartoum state/Sudan. BMC Res Notes. (2015) 8:502. doi: 10.1186/s13104-015-1509-x, PMID: 26419536 PMC4589074

[16] Sarmento LR Fernandes PFCBC Pontes MX Correia DBS Chaves VCB Carvalho CFA. Prevalence of clinically validated primary causes of end-stage renal disease (ESRD) in a state Capital in Northeastern Brazil. J Bras Nefrol. (2018) 40:130–5. doi: 10.1590/2175-8239-jbn-3781, PMID: 29782632 PMC6533992

[17] Pscheidt C Nagel G Zitt E Kramar R Concin H Lhotta K. Sex-and time-dependent patterns in risk factors of end-stage renal disease: a large Austrian cohort with up to 20 years of follow-up. PLoS One. (2015) 10:e0135052. doi: 10.1371/journal.pone.0135052, PMID: 26322515 PMC4555650

[18] Vinhas J Gardete-Correia L Boavida JM Raposo JF Mesquita A Fona MC. Prevalence of chronic kidney disease and associated risk factors, and risk of end-stage renal disease: data from the PREVADIAB study. Nephron Clin Pract. (2011) 119:c35–40. doi: 10.1159/000324218, PMID: 21654181

[19] Joyce Fan Z Lackland DT Lipsitz SR Nicholas JS Egan BM Tim Garvey W. Geographical patterns of end-stage renal disease incidence and risk factors in rural and urban areas of South Carolina. Health Place. (2007) 13:179–87. doi: 10.1016/j.healthplace.2005.12.002 16443385

[20] Khan S Hussain T Salahuddin N Mehreen S. Risk factors of end stage renal disease in Peshawar, Pakistan: odds ratio analysis. Open Access Maced J Med Sci. (2016) 4:381–7. doi: 10.3889/oamjms.2016.068, PMID: 27703559 PMC5042619

[21] Hsu CY Iribarren C McCulloch CE Darbinian J Go AS. Risk factors for end-stage renal disease: 25-year follow-up. Arch Intern Med. (2009) 169:342–50. doi: 10.1001/archinternmed.2008.605, PMID: 19237717 PMC2727643

[22] Crews DC Liu Y Boulware LE. Disparities in the burden, outcomes, and care of chronic kidney disease. Curr Opin Nephrol Hypertens. (2014) 23:298–305. doi: 10.1097/01.mnh.0000444822.25991.f6, PMID: 24662984 PMC4126677

[23] Bello AK Peters J Rigby J Rahman AA El Nahas M. Socioeconomic status and chronic kidney disease at presentation to a renal service in the United Kingdom. Clin J Am Soc Nephrol. (2008) 3:1316–23. doi: 10.2215/CJN.00680208, PMID: 18579673 PMC2518794

[24] Choi HS Han KD Jung JH Kim CS Bae EH Ma SK. The risk of end-stage renal disease in systemic lupus erythematosus: a nationwide population-based study in Korea. Medicine (Baltimore). (2019) 98:e16420. doi: 10.1097/MD.0000000000016420, PMID: 31305460 PMC6641670

[25] Plantinga L Lim SS Bowling CB Drenkard C. Association of age with health-related quality of life in a cohort of patients with systemic lupus erythematosus: the Georgians organized against lupus study. Lupus Sci Med. (2016) 3:e000161. doi: 10.1136/lupus-2016-000161 27547440 PMC4964216

[26] Manger K Manger B Repp R Geisselbrecht M Geiger A Pfahlberg A. Definition of risk factors for death, end stage renal disease, and thromboembolic events in a monocentric cohort of 338 patients with systemic lupus erythematosus. Ann Rheum Dis. (2002) 61:1065–70. doi: 10.1136/ard.61.12.1065, PMID: 12429536 PMC1753955

[27] Wunnenburger S Schultheiss UT Walz G Hausknecht B Ekici AB Kronenberg F. Associations between genetic risk variants for kidney diseases and kidney disease etiology. Sci Rep. (2017) 7:13944. doi: 10.1038/s41598-017-13356-6 29066732 PMC5655008

[28] Hsu CY McCulloch CE Iribarren C Darbinian J Go AS. Body mass index and risk for end-stage renal disease. Ann Intern Med. (2006) 144:21–8. doi: 10.7326/0003-4819-144-1-200601030-0000 16389251

[29] Locke JE Reed RD Massie A MacLennan PA Sawinski D Kumar V. Obesity increases the risk of end-stage renal disease among living kidney donors. Kidney Int. (2017) 91:699–703. doi: 10.1016/j.kint.2016.10.014, PMID: 28041626 PMC5313335

[30] Kastarinen M Juutilainen A Kastarinen H Salomaa V Karhapää P Tuomilehto J. Risk factors for end-stage renal disease in a community-based population: 26-year follow-up of 25,821 men and women in eastern Finland. J Intern Med. (2010) 267:612–20. doi: 10.1111/j.1365-2796.2009.02197.x, PMID: 20210838

[31] Li S McAlpine DD Liu J Li S Collins AJ. Differences between blacks and whites in the incidence of end-stage renal disease and associated risk factors. Adv Ren Replace Ther. (2004) 11:5–13. doi: 10.1053/j.arrt.2003.10.005 14730534

[32] Lebov JF Engel LS Richardson D Hogan SL Sandler DP Hoppin JA. Pesticide exposure and end-stage renal disease risk among wives of pesticide applicators in the agricultural health study. Environ Res. (2015) 143:198–210. doi: 10.1016/j.envres.2015.10.002, PMID: 26505650 PMC4662544

[33] Nakhoul GN Huang H Arrigain S Jolly SE Schold JD Nally Jr JV. Serum potassium, end-stage renal disease and mortality in chronic kidney disease. Am J Nephrol. (2015) 41:456–63. doi: 10.1159/000437151, PMID: 26228532 PMC4686260

[34] Sommar JN Svensson MK Björ BM Elmståhl SI Hallmans G Lundh T. End-stage renal disease and low level exposure to lead, cadmium and mercury; a population-based, prospective nested case-referent study in Sweden. Environ Health. (2013) 12:9. doi: 10.1186/1476-069X-12-9, PMID: 23343055 PMC3566948

[35] Aksoy N Şelimen D. Investigation of the causes and risk factors of previous end-stage renal disease in kidney transplant recipients. Transplant Proc. (2020) 52:140–5. doi: 10.1016/j.transproceed.2019.11.019, PMID: 31901330

[36] Østergaard HB Westerink J Verhaar MC Bots ML Asselbergs FW de Borst GJ. End-stage kidney disease in patients with clinically manifest vascular disease; incidence and risk factors: results from the UCC-SMART cohort study. J Nephrol. (2021) 34:1511–20. doi: 10.1007/s40620-021-00996-1, PMID: 33713332 PMC8494654

[37] Ruiz-Martínez A Sierra-Díaz E Celis-de la Rosa AJ Valenzuela Hernández MÁ González Flores MG Belmonte Hernández MV. Renal Doppler ultrasound resistive index vs. renal scintigraphy with 99mTc-DTPA as diagnostic test for ureteropelvic junction obstruction in children. Actas Urol Esp (Engl Ed). (2019) 43:419–24. doi: 10.1016/j.acuro.2019.02.005, PMID: 31164308

[38] Furlano M Arlandis R del Prado Venegas M Novelli S Crespi J Bullich G. MYH9 associated nephropathy. Nefrologia (Engl Ed). (2019) 39:133–40. doi: 10.1016/j.nefro.2018.08.008 30471777

[39] Sierra-Diaz E Gaxiola-Perez E Beas-Ruiz Velasco C Sedano-Portillo I Gonzalez-Gonzalez CA Adel-Dominguez M. Exposure to radioactive emanations of medical personnel in percutaneous Nephrolithotomy. Dose Response. (2018) 16:7930. doi: 10.1177/1559325818777930 PMC5974570 29872370

[40] Dávila-Radilla F Sierra-Díaz E Ochoa-Vega ME Gaxiola-Pérez E Beas Ruiz Velasco C Sedano-Portillo I. Frequency of fever after percutaneous nephrolithotomy: comparison between two prophylaxis schemes. Bol Col Mex Urol. (2020) 35:1–6.

[41] Sierra-Diaz E Celis-De la Rosa A Beas-Ruiz Velasco C. Incidence of fever and bleeding after percutaneous nephrolithotomy: a prospective cohort study. Cir Cir. (2021) 90:57–63. doi: 10.24875/CIRU.20001130 35120110

[42] Dahnan M Assabri AM Khader YS. Risk factors for end-stage renal failure among patients on hemodialysis in Aljomhory hospital, Sa’adah governorate, Yemen: hospital-based case-control study. JMIR Public Health Surveill. (2019) 5:e14215. doi: 10.2196/14215 31573930 PMC6785724

[43] Ingrasciotta Y Sultana J Giorgianni F Fontana A Santangelo A Tari DU. Association of individual non-steroidal anti-inflammatory drugs and chronic kidney disease: a population-based case control study. PLoS One. (2015) 10:e0122899. doi: 10.1371/journal.pone.0122899, PMID: 25880729 PMC4399982

[44] Pan Y Zhang L Wang F Li X Wang H. China National Survey of chronic kidney disease working group. Status of non-steroidal anti-inflammatory drugs use and its association with chronic kidney disease: a cross-sectional survey in China. Nephrology (Carlton). (2014) 19:655–60. doi: 10.1111/nep.12318, PMID: 25196389

[45] Ibáñez L Morlans M Vidal X Martínez MJ Laporte JR. Case-control study of regular analgesic and nonsteroidal anti-inflammatory use and end-stage renal disease. Kidney Int. (2005) 67:2393–8. doi: 10.1111/j.1523-1755.2005.00346.x, PMID: 15882284

[46] Kuo HW Tsai SS Tiao MM Liu YC Lee IM Yang CY. Analgesic use and the risk for progression of chronic kidney disease. Pharmacoepidemiol Drug Saf. (2010) 19:745–51. doi: 10.1002/pds.1962 20582905

[47] Gooch K Culleton BF Manns BJ Zhang J Alfonso H Tonelli M. NSAID use and progression of chronic kidney disease. Am J Med. (2007) 120:280.e1–7. doi: 10.1016/j.amjmed.2006.02.015 17349452

[48] Højlund M Lund LC Herping JLE Haastrup MB Damkier P Henriksen DP. Second-generation antipsychotics and the risk of chronic kidney disease: a population-based case-control study. BMJ Open. (2020) 10:e038247. doi: 10.1136/bmjopen-2020-038247, PMID: 32784262 PMC7418669

[49] Perneger TV Klag MJ Whelton PK. Recreational drug use: a neglected risk factor for end-stage renal disease. Am J Kidney Dis. (2001) 38:49–56. doi: 10.1053/ajkd.2001.25181, PMID: 11431181

[50] Massie AB Holscher CM Henderson ML Fahmy LM Thomas AG al Ammary F. Association of early Postdonation renal function with subsequent risk of end-stage renal disease in living kidney donors. JAMA Surg. (2020) 155:e195472. doi: 10.1001/jamasurg.2019.5472, PMID: 31968070 PMC6990748

[51] Lange J Peterson SM Takashima JR Grigoriev Y Ritchey ML Shamberger RC. Risk factors for end stage renal disease in non-WT1-syndromic Wilms tumor. J Urol. (2011) 186:378–86. doi: 10.1016/j.juro.2011.03.110, PMID: 21683387 PMC3133859

[52] Shlipak MG Tummalapalli SL Boulware LE Grams ME Ix JH Jha V. The case for early identification and intervention of chronic kidney disease: conclusions from kidney disease: improving global outcomes (KDIGO) controversies conference. Kidney Int. (2021) 99:34–47. doi: 10.1016/j.kint.2020.10.012, PMID: 33127436

[53] Polanco-Flores NA. Chronic renal disease in Mexico: a preventive uncontrolled epidemic. Rev med Hosp Gen Méx. (2019) 82:194. doi: 10.24875/hgmx.m19000030

[54] Komenda P Ferguson TW Macdonald K Rigatto C Koolage C Sood MM. Cost-effectiveness of primary screening for CKD: a systematic review. Am J Kidney Dis. (2014) 63:789–97. doi: 10.1053/j.ajkd.2013.12.012, PMID: 24529536

[55] Rosa-Diez G Gonzalez-Bedat M Ferreiro A García-García G Fernandez-Cean J Douthat W. Burden of end-stage renal disease (ESRD) in Latin America. Clin Nephrol. (2016) 86:29–33. doi: 10.5414/CNP86S105 27509582

[56] Gutierrez-Peña M Zuñiga-Macias L Marin-Garcia R Ovalle-Robles I García-Díaz AL Macías-Guzmán MJ. High prevalence of end-stage renal disease of unknown origin in Aguascalientes Mexico: role of the registry of chronic kidney disease and renal biopsy in its approach and future directions. Clin Kidney J. (2021) 14:1197–206. doi: 10.1093/ckj/sfaa229, PMID: 34094519 PMC8173605

[57] Sayin A Mutluay R Sindel S. Quality of life in hemodialysis, peritoneal dialysis, and transplantation patients. Transplant Proc. (2007) 39:3047–53. doi: 10.1016/j.transproceed.2007.09.030 18089319

[58] Kimmel PL Patel SS. Quality of life in patients with chronic kidney disease: focus on end-stage renal disease treated with hemodialysis. Semin Nephrol. (2006) 26:68–79. doi: 10.1016/j.semnephrol.2005.06.015 16412831

[59] Adejumo OA Iyawe IO Akinbodewa AA Abolarin OS Alli EO. Burden, psychological well-being and quality of life of caregivers of end stage renal disease patients. Ghana Med J. (2019) 53:190–6. doi: 10.4314/gmj.v53i3.2, PMID: 31741490 PMC6842729

[60] Zachariah MS Dwivedi AK Yip CS Chang SS Gundroo A Venuto RC. Utility of serial protocol biopsies performed after 1 year in predicting long-term kidney allograft function according to histologic phenotype. Exp Clin Transplant. (2018) 16:391–400. doi: 10.6002/ect.2016.0323, PMID: 29206090

[61] Garcia-Garcia G Chavez-Iñiguez JS. The tragedy of having ESRD in Mexico. Kidney Int Rep. (2018) 3:1027–9. doi: 10.1016/j.ekir.2018.07.018 30197968 PMC6127446

[62] Mathur AK Ashby VB Sands RL Wolfe RA. Geographic variation in end-stage renal disease incidence and access to deceased donor kidney transplantation. Am J Transplant. (2010) 10:1069–80. doi: 10.1111/j.1600-6143.2010.03043.x, PMID: 20420653

[63] Gordillo R Munshi R Monroe EJ Shivaram GM Smith JM. Benefits and risks of protocol biopsies in pediatric renal transplantation. Pediatr Nephrol. (2019) 34:593–8. doi: 10.1007/s00467-018-3959-6, PMID: 29725772

[64] Zotta F Guzzo I Morolli F Diomedi-Camassei F Dello Strologo L. Protocol biopsies in pediatric renal transplantation: a precious tool for clinical management. Pediatr Nephrol. (2018) 33:2167–75. doi: 10.1007/s00467-018-4007-2, PMID: 29980849

[65] Böhmig GA Regele H Hörl WH. Protocol biopsies after kidney transplantation. Transpl Int. (2005) 18:131–9. doi: 10.1111/j.1432-2277.2004.00020.x 15691264

[66] Sellarés J de Freitas DG Mengel M Reeve J Einecke G Sis B. Understanding the causes of kidney transplant failure: the dominant role of antibody-mediated rejection and non-adherence. Am J Transplant. (2012) 12:388–99. doi: 10.1111/j.1600-6143.2011.03840.x, PMID: 22081892

[67] Infante B Rossini M Leo S Troise D Netti GS Ranieri E. Recurrent glomerulonephritis after renal transplantation: the clinical problem. Int J Mol Sci. (2020) 21:5954. doi: 10.3390/ijms21175954 32824988 PMC7504691

[68] Kidney Disease: Improving Global Outcomes. CKD work group KDIGO 2 clinical practice guideline for the evaluation and management of chronic kidney disease. Kidney Int Suppl. (2013) 3:1–150. doi: 10.1038/kisup.2012.73

