# Supplementary material for: Non-traditional risk factors of progression of chronic kidney disease in adult population: a scoping review
Source: Front Med (Lausanne). 2023 Jun 2;10:1193984. doi: 10.3389/fmed.2023.1193984 (PMC10272583; doi:10.3389/fmed.2023.1193984)
Supplement: Supplementary file 1 [file Data_Sheet_1.pdf]

Supplementary Material

NON-TRADITIONAL RISK FACTORS OF END-STAGE RENAL DISEASE IN ADULT POPULATION: A SCOPING REVIEW

Diana Lorena Cisneros-García1, Elena Sandoval-Pinto2*, Rosa Cremades 3, Adrián Ramírez – de-Arellano 4, Mariana García-Gutiérrez5, Roberto Martínez-de-Pinillos-Valverde6, Erick Sierra-Díaz,7, 8.

*** Correspondence:** Erick Sierra-Díaz M.D, Ph.D.

erksland@hotmail.com

1 Supplementary Figures and Tables

2 Table 1. PubMed used words and number of hints

	Key-Word combination	Number of Hints	Final Selection
PubMed	End-stage renal disease OR End-Stage Kidney Disease OR Chronic Kidney Failure OR Chronic Renal Failure OR End-Stage Renal Failure AND risk factors OR causes	200	43
PubMed	End-stage renal disease OR End-Stage Kidney Disease OR Chronic Kidney Failure OR Chronic Renal Failure OR End-Stage Renal Failure AND risk factors OR causes AND unknown OR non-traditional	17	0<br>(3 repeated)
PubMed	End-stage renal disease OR End-Stage Kidney Disease OR Chronic Kidney Failure OR Chronic Renal Failure OR End-Stage Renal Failure AND risk factors OR causes NOT diabetes	126	3<br>(7 repeated)
PubMed	End-stage renal disease OR End-Stage Kidney Disease OR Chronic Kidney Failure OR Chronic Renal Failure OR End-Stage Renal Failure AND risk factors OR causes NOT hypertension	133	0<br>(10 repeated)
	Total	476	46

3 Table 2. Characteristics of the studies included

AUTHOR (YEAR) COUNTRY [REF]	OBJECTIVE	STUDY DESIGN	SAMPLE	VARIABLES	STATICAL ANALYSIS	RESULTS
**Vinhas, J (2011) Portugal [18]**	To evaluate the prevalence of diabetes, was an opportunity to evaluate the prevalence of CKD stages 3 and 4 and associated risk factors in Portugal	Cross-sectional	n=5,167	ESRD, age, gender, educational level, Family background and history of hypertension. The clinical examination included blood pressure measurement, waist circumference, weight, and height blood	Logistic regression for multiple variables.	In 2008, there were 47 new ESRD patients for each 10,000 patients with CKD stages 3 and 4. In this study, the risk of ESRD was greater among men. Annually, the risk of ESRD was 109 in males and 25 in females for each 10,000 patients with CKD stages 3 and 4.
**Fan, Z (2005) USA [19]**	To determine the spatial distribution of ESRD incidence using geographical information systems (GIS); and second, to evaluate the ecologic association between the county level risk factors and incidence of ESRD	Ecological	n=11,646	ESRD, date of birth, date of diagnosis, race, sex, and county of residence at the time of diagnosis, diabetes, hypertension, population physician density, and SES indicators	Chi-squared test and student's t-test. Using linear regression, A Poisson model was constructed for multivariate analysis.	African Americans had higher risk of ESRD incidence rates compared to Caucasians (adjusted relative risk (RR) 2.86, 95% confidence interval (95% CI) 2.35–3.47) after adjusting age, gender and county of residence.
**Khan, S (2016) Pakistan [20]**	To discover the association of End Stage Renal Disease	Cross-sectional	n=407	ESRD, Gender, diabetic, hypertension, glomerulonephritis, polycystic kidney disease, myeloma,	The association of various risk factors with the occurrence of ESRD was determined	The odds of ESRD show that the males are 1.4 times more exposed to ESRD than the females

	(ESRD) with various risk factors.			SLE nephritis, hepatitis, heredity, drug usage, heart problem, anemia	through the statistical technique of odds ratio analysis	
**Hsu, CY (2009) USA** [21]	To evaluate the prognostic value of several potential novel risk factors for ESRD after considering<br><br>established risk factors	Cohort	n=177,570	ESRD, risk factors were further divided into the following 4 groups: clinical, family history, occupational exposure, and behavioral.	Cox proportional hazards model. A likelihood ratio statistic. Multivariate model.	African American race (HR, 3.02 [95% CI, 2.58–3.54] vs white race.<br><br>selected occupational exposures (to lead or other metal fumes [HR, 1.85 (CI, 1.44–2.38)]; asbestos, cement or grain dust [HR, 1.77 (CI, 1.34–2.33)]; ammonia, chlorine, ozone or nitrous gas [HR, 1.71 (CI, 1.37–2.15)]; chemicals, cleaning fluid or solvents [HR, 1.61 (CI, 1.37–1.90)]; engine exhaust fumes [>2 h/d] [HR, 2.00 (CI, 1.62–2.46)]; extreme heat [HR, 1.73 (CI, 1.34–2.24)]; insect or plant spray [HR, 1.78 (CI, 1.36–2.34)]; silica, sandblasting, grinding or rock dust) [HR, 1.53 (CI, 1.15–2.05)];
**Crews (2014) USA** [22]	To examine associations of income inequality and residence, as social determinants of health, with survival among black and white patients with ESRD	Cohort	n=589,036	Age, year of dialysis initiation, race, sex, BMI (kg/m2), current smoking status, insurance, and 6-month prior employment status, as well as primary cause of renal failure and comorbid conditions.	Cox proportional hazards regression analyses	Middle income level subjects showed lower risk (1.0 95CI 1.01-1.06) and those individuals with high incomes reported HR values <1.
**Bello, AK (2005) UK** [23]	To study the association between area-level	Cross-sectional	N=1657	Progression of chronic kidney disease, low socioeconomic	Binary logistic regression analysis	In the non-Caucasian patients, those in the fifth IMD quintile carried the greatest risk (RR 9.33; 95% CI 1.03 to 84.20), a risk

	SES and severity of established CKD at presentation to a renal service in the United Kingdom and also examined whether any association is independent of established risk factors for CKD.			status		higher than the corresponding category in the Caucasian population
**Choi, HS (2019) Korea** [24]	To investigate the incidence rate of ESRD and risk factors for progression to ESRD in Korean SLE patients compared to the general population.	Cohort	n=21, 253	ESRD, diabetes, hypertension, dyslipidemia, myocardial infarction, presence of stroke, creatinine, glomerular filtration rate	Kaplan-Meier curves, Cox proportional hazard models-Multivariable-adjusted proportional hazard models, survival analysis	In the subgroup analysis, male (HR, 7.76; 95% CI, 5.07–11.90) and female patients (HR, 10.479; 95% CI, 8.405–13.066) with SLE had a higher risk of ESRD than the non-SLE matched controls. The risk for ESRD was higher in 20 to 39-year-old patients with SLE (HR, 22.41; 95% CI 14.20–35.36), 40–64-year-old patients with SLE (HR, 8.46; 95% CI 6.42–11.13), and ≥65-year-old patients with SLE (HR, 5.01; 95% CI 3.34–7.53) than in matched controls.
**Plantinga, L (2016) USA** [25]	To examine whether older age was associated with lower health-related quality of life (HRQOL) among patients with systemic lupus	Cohort	n=71 4	ESRD, SLE, age at SLE onset, sex, race, ethnicity, body mass index (BMI), education, employment status, income and marital status	Multivariable linear regression models	Systemic Lupus Erythematosus (SLE) is reported as an important direct cause for ESRD (HR 6.7 95CI 2.6-16)

	erythematosus (SLE) and whether differential disease-related damage and activity explained these associations.					
**Manger, K (2002) Germany [26]**	To identify new risk factors for a severe outcome<br><br>in SLE, as defined by death, ESRD, and thromboembolic<br><br>events (TE).	Cohort	n=338	ESRD, lupus with low C3, splenomegaly or Nephritis (ACR)	Kaplan-Meier curves Multivariable Cox regression models	We found no significant sex differences in 4% of patients<br><br>with ESRD in the whole SLE cohort, however, a low C3<br><br>(RR=3.0, 95% CI 1.5 to 6.0, p<0.01)
**Wunnenburger S (2017) Germany [27]**	To examine whether risk loci discovered in the general population are also associated with advanced stages of CKD and with CKD in patients for whom the leading cause of disease was hypertension or diabetes, the most common causes of CKD.	Prospective observational study	n=5,217	Cause of CKD, Serum creatinine, regardless of CKD etiology	Multivariable adjusted logistic regression analyses	SLE risk variant rs1150754 at TNXB was associated with CKD attributed to T1DM (OR = 2.53, p = 2.5 × 10−7)

**Hsu, CY (2006) USA** [28]	To determine the association between increased body mass index (BMI) and risk for ESRD	Cohort	n=320,252	ESRD<br><br>body mass index (BMI)<br><br>sex, race, age,<br><br>diabetes mellitus, hypertension, and presence or absence of<br><br>baseline kidney disease.	multivariable analyses by using Cox proportional<br><br>hazards models	Compared with persons who had normal weight (BMI, 18.5 to 24.9 kg/m2), the adjusted relative risk for ESRD was 1.87 (95% CI, 1.64 to 2.14) for those who were overweight (BMI, 25.0 to 29.9 kg/m2), 3.57 (CI, 3.05 to 4.18) for those with class I obesity (BMI, 30.0 to 34.9 kg/m2), 6.12 (CI, 4.97 to 7.54) for those with class II obesity (BMI, 35.0 to 39.9 kg/m2), and 7.07 (CI, 5.37 to 9.31) for those with extreme obesity (BMI ≥ 40 kg/m2)
**Locke, JE (2008) USA** [29]	To study of the association between BMI and post-donation risk of ESRD among living kidney donors, adjusting for potential confounders and exploring potential effect modifiers of this association.	Cohort	n=2,013	ESRD<br><br>BMI, pre-operative systolic and diastolic blood pressure, baseline eGFR, insurance status, relationship to the recipient, and smoking status	Kaplan-Meier method, survival analyses	Compared to non-obese living kidney donors, obese living kidney donors had a 1.9-fold increased risk of ESRD post-donation (adjusted hazard ratio (aHR): 1.86, 95%CI: 1.05–3.30, p=0.04)
**Kastarinen, M (2009) Finland** [30]	To assess whether the role of classical, life-style related chronic disease risk factors in predicting ESRD is similar in a large	Cohort	n=28,821	ESRD<br><br>Medical history, health behaviour and socio-economic factors, physical activity, diagnosis of diabetes, smoking status, blood pressure (BP), height and	hazards regression model	The association between BMI and ESRD was similar as in comparison between obese and normal weight persons when we used quartile cut-offs for BMI [hazard ratio (HR) for the highest vs. the lowest quartile 2.63, 95% confidence interval (CI) 1.45–4.76, data not shown].

	community based sample of men and women in Eastern Finland as in previous studies done in other parts of the world.			weight.		
**Li, S (2004) USA** [31]	To investigated risk factors associated with the higher incidence of ESRD in blacks compared with whites in Medicare patients 65 years old and older.	Retrospective follow-up	n=1,055,236	ESRD, age and gender, socioeconomic status, special health conditions, primary causal diseases of ESRD, diabetes care and preventive care, and physician visits for primary or specialty care	Cox regression	Patients with a diagnosis of anemia (RR, 2.81; P < .0001) or cardiovascular disease (RR, 1.55; P < .0001) were more likely to develop ESRD; however, those with chronic obstructive pulmonary disease (RR, 0.88; P = .0033) were less likely to develop ESRD.
**Khan, S (2016) Pakistan** [32]	To discover the association of End Stage Renal Disease (ESRD) with various risk factors.	Cross-sectional	n=407	ESRD, gender<br><br>Diabetic<br><br>Hypertension<br><br>Glomerulonephritis<br><br>Polycystic kidney disease<br><br>Myeloma<br><br>SLE nephritis<br><br>Hepatitis<br><br>Heredity<br><br>Drug usage<br><br>Heart problem<br><br>Anemia	Univariate and Cox regression analysis	ESRD versus anemia. The calculated values are: Odd Ratio = 0.088, p-value = 0.788 and Confidence Interval is (0.676, 4.024).

**Lebov, JF (2015) USA** [33]	To examine rates of ESRD among wives in relation to their personal use of specific pesticides and pesticide use by their applicator husbands. We also evaluated the association between other non-application pesticide exposure opportunities and ESRD.	Cohort	n=32, 346	ESRD, use of 50 pesticides and duration and frequency of use for 22 of those pesticides	Cox proportional hazards models	ESRD was positively associated with ever use of alachlor for both direct (wives' personal use: HR = 1.85; 95% CI: 0.67, 5.12) and indirect exposures (husbands' use: HR = 1.63; 95% CI: 0.91, 2.91). The rate of ESRD was elevated in association with direct, but not indirect, exposure to the herbicides chlorimuron-ethyl (HR = 4.03; 95% CI: 1.30, 12.51; indirect) and imazethapyr (HR = 2.37; 95% CI: 0.76, 7.36;), though only three cases reported using each of these pesticides.
**Nakhoul, GN (2015) USA** [34]	To examine the associations between serum potassium and all-cause mortality and ESRD in a cohort of stage 3 and stage 4 CKD patients following in our health care system.	Cross-sectional	n=15 2,009	ESRD, serum potassium Diabetes mellitus, hypertension, coronary artery disease, and other comorbid conditions	Logistic regression analyses, Cox proportional hazards models	High serum potassium levels (>5.0 mmol/l) In the multivariable analysis, the following variables were associated with higher odds of having high potassium levels compared to normal: male gender, lower eGFR, BMI <18.5 kg/m2, diabetes, malignancy, and use of ACE/ARB.
**Sommar, JN (2013) Sweden** [35]	To elucidate how erythrocyte concentrations of Pb (Ery-Pb),	Cohort	n=30 ,447	ESRD, smoking, blood pressure, diabetes, and hypertension	Univariate analyses, Spearman's correlation coefficient, multiple	The OR for one µg/L increase of Ery-Pb was 1.013 (95% CI 1.003-1.023) and for Ery-Hg 0.81 (95% CI 0.668-0.99), when also taking into consideration

	Cd (Ery-Cd), and Hg (Ery-Hg) in the general population relate to the risk of developing end-stage renal disease later in life.				conditional logistic regression model	diabetes, BMI and hypertension.
**Aksoy, N (2020) Turkey** [36]	To determine the causes and risk factors of previous ESRD in kidney transplant (KT) recipients.	Cross-sectional.	n=450	ESRD, demographic characteristics, diseases,<br><br>treatments they underwent, their genetic characteristics, environmental<br><br>characteristics, habits, and lifestyles	Pearson x2 test, or Fisher exact x2 test, Spearman correlation analysis logistic regression, multivariate analysis	In patients who reported stressed levels before ESRD was5.86 times higher (OD: 5.86; P ¼ .000; 95% CI [2.212-15.528]) compared to patients who reported little to no stress.
**Østergaard, HB (2021) Netherlands** [37]	To determine the incidence of ESKD in patients with stable manifest CVD according to vascular disease location, and to assess the relation between modifiable risk factors for kidney disease and incident ESKD in a contemporary population cohort with stable	Cohort	n=8,402	End-stage kidney disease (ESKD).<br><br>Glomerular filtration rate (eGFR), systolic blood pressure (SBP), t2DM, non-HDL-cholesterol, smoking, exercise.	Cox proportional hazard models	Using the model with clinical covariates, current smoking was independently associated with an elevated risk of ESKD (HR 1.87; 95% CI 1.10–3.19)

	manifest vascular disease					
**Ruiz-Martínez A (2019) México** [38]	To evaluating the usefulness of Doppler ultrasound with resistive index (RI) measure compared with renal scintigraphy with 99m Tc-DTPA in children with unilateral ureteropelvic junction obstruction	Cross-sectional	n=21	ESRD, doppler ultrasound, Tc-DTPA, unilateral ureteropelvic junction obstruction	t-test analysis, fisher statistical test	Ureteropelvic junction obstruction is the most common congenital abnormality diagnosed in children. However, in many cases, this congenital abnormality is diagnosed in adults being unilateral combined with other chronic diseases or bilateral in some cases causing ESRD
**Furlano M, (2019) Barcelona** [39]	To show an example of the delayed diagnosis of uncommon diseases and highlights the usefulness genetic testing.	Genetic study	n=1	ESRD, MYH9 gene, Alport syndrome	correlation genotype-phenotype	In our case, the patient presented a mutation<br><br>in exon 2 of the MYH9 gene (c.287C>T), which determine the<br><br>substitution of the amino acid serine for leucine (p.Ser96Leu)<br><br>in the N-terminal domain of the protein
**Sierra-Diaz E (2018) Mexico** [40]	To measure the extent of radiation exposure in surgeons performed during PNLs in patients with ventral decubitus position with double	Cross-sectional	n=34	Exposure time (sec) to radiation, surgery time	Univariate, descriptive statistics and central trend measurements.	Regarding kidney stone density, average density was 1005.7 Hounsfield Units (HU), with ranges of 453 to 1800 HU. Average fluoroscopy time was 58.3 seconds (range, 24-122 seconds) in both groups; the average for males was 57.16 seconds per case, while that for females was 58.95 seconds (P = .6). Total fluoroscopy time emitted by the fluoroscope during the procedures was 1983

	flexion					seconds (equivalent to 33.05 minutes)
**Dávila-Radilla F (2020) México** [41]	To measure the frequency<br><br>of fever after percutaneous nephrolithotomy using two types of prophylactic antibiotic scheme	Cross-sectional	n=119	Age in years, sex<br><br>height (meters), weight (kg), BMI, stone size (cm3), essence (HU), fever	Mann-Whitney U test and bivariate analysis (OR)<br><br>was calculated using cross-tabulation	Cross-tabulation analysis was used to compare<br><br>the results between group 1 and 3 (OR = 4.4,<br><br>95%CI [9.47-40.5] p>0.05). The same method<br><br>was used to compare group 2 and 3 (OR = 3.1,<br><br>95%CI [0.37-26.6] p>0.05), and for group 1<br><br>versus group 2 (OR = 1.4, 95%CI [0.41-4.7]<br><br>p>0.05). Comparison between the infected and<br><br>the total non-infected (groups 2 and 3) yielded<br><br>results (OR = 1.7, 95%CI [0.5-5] p>0.05)
**Sierra-Diaz E (2022) México** [42]	To report the incidence of complications during and after PCNL based on a cohort study design.	Cohort	n=101	Age (years), eeight (Kilograms), height (meters), corporal Mass Index, exposed (positive urine culture).	univariate methods, bivariate analysis	In general terms, total incidence of complications for both groups was 19%. The exposed group presented complications in 27%, while complications were found in 16% of the non-exposed group. RR of complications in general was 1.68 (95% CI, 0.77-3.6), AR was 11.09, and PAR was 40.68 of complications
**Dahnan, M (2019) Yemen** [43]	To determine the risk factors of ESRF in Sa'adah Governorate in Yemen	Case-control	n=349	ESRD<br><br>Age<br><br>Education<br><br>Gender	Chi-square test independent t test. Binary logistic regression.	In multivariate analysis, hypertension (OR=6.7), presence of urinary stones (OR=16.1), and recurrent urinary tract infection (OR=8.7) were the only factors associated with ESRD after adjusting for other variables.

				Hypertension<br><br>Diabetes mellitus<br><br>Cardiovascular study<br><br>disease<br><br>Urinary stones<br><br>Recurrent urinary tract infection<br><br>Cigarette smoking<br><br>Shammah use		
**Ingrasciotta Y (2015) Italy** [44]	To evaluate and compare the association between the use of individual NSAIDs and risk of CKD in a general population of Southern Italy.	Cross-sectional	n=15 8,510	CKD, potential risk factors for CKD, including co-morbidities (i.e.<br><br>malignant neoplasm, ischemic heart disease, diabetes mellitus, liver disease, gout, dyslipidemia,<br><br>hypertension, cerebrovascular disease, lupus erythematosus systemic (LES), amyloidosis, vasculitis, myeloma, polycystic kidney disease), and prior use of known nephrotoxic drugs other<br><br>than NSAIDs (e.g. aminoglycosides, gold preparations, and lithium)	Univariate and multivariate conditional logistic regression	A statistically significant increase in the CKD<br><br>risk was found for current users of oxicams (adj. OR: 1.74; 95% CI: 1.20–2.54; p = 0.004) and,<br><br>concerning single ingredients, for current users of meloxicam (adj. OR: 1.98; 95% CI: 1.01–<br><br>3.87; p = 0.046), piroxicam (adj. OR: 1.95; 95% CI: 1.19–3.21; p = 0.008) and ketorolac (adj.<br><br>OR: 2.54; 95% CI: 1.45–4.44; p = 0.001). No statistically significant associations were found for<br><br>larger time windows
**Pan Y (2012) China** [45]	To measure the prevalence of chronic	Cross-sectional	n=47, 204	CDK, sex, history of<br><br>cardiovascular	multivariable logistic regression	OR values for sex and age, resulting in eGFR <60 ml/min per 1.73 m2 (OR 1.51 CI95 1 .13-2.02), and

	kidney disease in China with such a survey.			disease, hypertension,<br><br>diabetes, education, smoker, alcohol intake,<br><br>hepatitis B virus infection, nephrotoxic medications, hyperuricaemia, plasma triglyceride,<br><br>plasma LDL cholesterol, plasma HDL cholesterol		albuminuria (OR 1.31 CI95 1.07-1.59). Same authors identified that the cumulative time of NSAID intake and kidney damage for those who took them for more than 48 months had an eGFR <60 ml/min per 1.73 m2 (OR 2.36 (CI95 1 .28-4.37)
**Ibáñez L (2005) Spain** [46]	To estimate the risk of ESRD associated with the chronic use of analgesics and NSAIDs	Case-control	N= 1,502	NSAID user, age, sex, number of years of education, detailed medical information including dates and other information on the first diagnosis of renal disease, first appearance of signs or symptoms of renal disease, and a history of major illnesses, occupational exposures, smoking, and alcohol and caffeine consumption.	Conditional or unconditional logistic models	Chronic use of analgesic drugs or NSAIDs (according to the predefined criteria) was reported by 137 cases (23.5%) and 196 controls (16.5%), thus giving an odds ratio of 1.22 (95% CI, 0.89–1.66)
**Kuo HW (2010) Taiwan** [47]	To clarify the renal risk of analgesic use in CKD patients.	Cohort	n= 19,163	CKD, ESRD, COX2 inhibitors, NSAIDs, Acetaminophen, Aspirin	Cox proportional hazard model	CKD patients using acetaminophen, aspirin, COX-2 inhibitors, and other NSAIDs had an increased risk of progression to ESRD with multivariable adjusted HR of 2.92 (CI95 2.47-3.45), 1.96 (CI95 1.62-2.36), 1.54 (CI95 1.08-2.20) and 1.56 (CI95 1.32-1.85), respectively
**Gooch K (2007) Canada** [48]	To determine if this association	Cohort	n= 10,184	CKD, age, sex, baseline estimated glomerular filtration rate	Multiple logistic regression analyses	COX-2 inhibitor users had a 25% increased risk of rapid progression of kidney disease (odds ratio [OR]

	differed for conventional nonselective NSAIDs versus selective COX-2 inhibitors.			(eGFR), diabetes, and comorbidity were used to explore the associations of NSAID		1.25, 95% confidence interval [CI], 1.05-1.47) and traditional NSAID users a 29% increased risk (OR 1.29, 95% CI, 1.02-1.63) compared with non-NSAID users. There was no association between NSAID use and rapid progression of chronic kidney disease for the other 2 categories of mean glomerular filtration rate
**Højlund M (2020) Denmark** [49]	To examine the association between use of second-generation antipsychotics (SGA) and the risk of chronic kidney disease (CKD).	Case-control	n= 107,010	CKD, age, sex and calendar time (accounted for by sampling procedure), (ii) use of other drugs known to affect renal function (lithium and non-steroidal anti-inflammatory drugs (NSAIDs)), (iii) history of hypertension and diabetes, and (iv) highest achieved level of education as a proxy for socioeconomic status	logistic regression	Among cases, 557 (2.6%) were ever users of SGAs compared with 1731 (2.0%) of controls, yielding an aOR of 1.24 (95%CI: 1.12 to 1.37). The corresponding aOR for current use was 1.26 (95%CI: 1.12 to 1.42).
**Perneger TV (2001) USA** [50]	To examine whether the use of street drugs is associated with an increased risk for treated ESRD in a general population	Case-control	n= 1,077	ESRD, age, sex, race, socioeconomic status, history of diabetes, history of hypertension, intake of analgesics, alcohol use	Cross-tabulation and the use of logistic regression.	ESRD in people who used cocaine less than 100 times in their lives (OR 2.6 IC951.2-6.5), compared to those people who used it more than 100 times in their lives (OR 8.7 CI95 2.2-75.3). Heroin use showed a strong and statistically significant incresed risk for ESRD (OR 20.3 CI95 3.6-infinite)
**Massie AB (2020) USA** [51]	To determine the association	Cohort	n= 71,468	Renal function as measured by estimated glomerular	Cox regression	Postdonation eGFR was associated with ESRD: stratified by eGFR6, the 15-year cumulative incidence of

	between renal function in the first 6 months postdonation and subsequent risk of ESRD in kidney donors.			filtration rate 6 months after donation (eGFR6).<br><br>End-stage renal disease.		ESRD ranged from 11.7 donors per 10 000 donors with eGFR6 values greater than 70 mL/min/1.73 m2 to 33.1 donors per 10 000 donors with eGFR6 values of 50 mL/min/1.73 m2 or less (P = .049)
**Lange, J (2011) USA** [52]	To examined the association between ESRD due to chronic renal failure (CRF) and other genital anomalies, specifically streak ovaries and disorders of sexual differentiation (DSD), that are associated with Wilms tumor (WT1) mutations and may represent a forme fruste of Denys-Drash (DDS).	Cohort	n=7,950	ESRD, age at WT Diagnosis<br><br>Histology<br><br>Nephrogenic Rests<br><br>Radiation Dose to Contralateral Kidney (Gy)<br><br>Denys-Drash (DDS)<br><br>Wilms tumor-aniridia (WAGR) Genitourinary Anomalies (GU)	Cox regression. Single and multiple regressions Tests of proportionality	Radiation of 15 Gy or more to the contralateral kidney was associated with an increased risk of ESRD, particularly of ESRD due to PBWT for which the HR was 4.2 (p=.003).
